# Supplementary material for: Selection of Soybean and Cowpea Cultivars with Superior Performance under Drought Using Growth and Biochemical Aspects
Source: Plants (Basel). 2023 Aug 31;12(17):3134. doi: 10.3390/plants12173134 (PMC10489739; doi:10.3390/plants12173134)
Supplement: Supplementary file 1 [file plants-12-03134-s001.zip › plants-2561890-supplementary.pdf]

plants

MDPI

Article

Selection of soybean and cowpea cultivars with superior performance under drought using growth and biochemical aspects

Rafael de Souza Miranda [1,2,*], Bruno Sousa Figueiredo da Fonseca [3], Davielson Silva Pinho [3]; Jennyfer Yara Nunes Batista [3]; Ramilos Rodrigues de Brito [2], Everaldo Moreira da Silva [3], Wesley Santos Ferreira [3], José Hélio Costa [4], Marcos dos Santos Lopes [3], Renan Henrique Beserra de Sousa [2], Larissa Fonseca Neves [3], José Antônio Freitas Penha [3], Amanda Soares Santos [2], Juliana Joice Pereira Lima [3], Stelamaris de Oliveira Paula-Marinho [2], Francisco de Alcântara Neto [1], Évelyn Silva de Aguiar [5], Clesivan Pereira dos Santos [5] and Enéas Gomes-Filho [4]

[1] Plant Science Department, Federal University of Piauí, Teresina, Piauí, Brazil; fneto@ufpi.edu.br
[2] Postgraduate Program in Agricultural Sciences, Campus Professora Cinobelina Elvas, Federal University of Piauí, Bom Jesus, 64900-000, Piaui, Brazil; ramilos@hotmail.com, renanbiologiabomjesus@gmail.com, amandasantosagro@gmail.com, stelamarisop@live.com
[3] Agronomic Engineering Course, Campus Professora Cinobelina Elvas, Federal University of Piauí, Bom Jesus, 64900-000, Piaui, Brazil; brunofigueiredo91@ufpi.edu.br, davielson5@gmail.com, jennyferyaranb@gmail.com, everaldo@ufpi.edu.br, wsferreira18@hotmail.com, marcossantos319@gmail.com, larissafonseca034@gmail.com, joseafpenha@outlook.com, julianalima@ufpi.edu.br
[4] Department of Biochemistry and Molecular Biology, Federal University of Ceará, Fortaleza 60451-970, Ceará, Brazil: helio.costa@ufc.br, egomesf@ufc.br
[5] Postgraduate Program in Environmental Sciences, Center of Sciences of Chapadinha, Federal University of Maranhão, Boa Vista, 65500-000, Chapadinha, Maranhão, Brazil; evellynas@outlook.com, clesivan.pereira@ufma.br
* Correspondence: rsmiranda@ufpi.edu.br

Supplementary material

Table S1. Soybeans cultivars grown in agricultural areas of the Brazilian Cerrado.

Soybean Cultivars	Number in NRC (1)	Beginning of protection	Relative Maturity Group	Type of growth
AS3810 IPRO	20170297	June 01st, 2017	8.1	Determined
M8644 IPRO	20150187	March 16th, 2015	8.6	Determined
TMG1180 RR	20160073	October 06th, 2015	8.0	Semi-determined
NS8338 IPRO	20170297	June 01st, 2017	8.3	Determined
BMX81I81 IPRO	WR(2)	-	8.1	Indetermined
M8808 IPRO	20160171	March 11th, 2016	8.8	Determined
BÔNUS8579 IPRO	WR	-	7.9	Indetermined

Note - (1) National Registry of Cultivars of the Ministry of Agriculture, Livestock, and Supply. (2)WR - Without registration.

Table S2. Cowpea cultivars grown in agricultural areas of the Brazilian semiarid region.

Cowpea Cultivars	Number in NRC (1)	Registration date (2)	Coordinating institution of release	Breeding method	Commercial subclass
Aracê	25892	August 26th, 2009	Embrapa Meio-Norte	Genealogical	Green
Novaera	22156	September 10th, 2007	Embrapa Meio-Norte	Genealogical	White
Pajeú	22995	April 11th, 2008	Embrapa Meio-Norte	Single-pod descent	Mulatto
Pitiúba(2)	5177	June 19th, 2000	UFC	Mass selection	Mulatto
Tumucumaque	22891	March 6th, 2008	Embrapa Meio-Norte	Single-pod descent	White
TVU	WR(3)	-	EMAPA(4)	Introduction and selection among accesses	Mulatto
Xique-xique	22997	April 11th, 2008	Embrapa Meio-Norte	Single-pod descent	White

Note - (1) National Registry of Cultivars of the Ministry of Agriculture, Livestock, and Supply. (2) Registered in the Access Registry Book of the Cowpea Germplasm Bank at the Federal University of Ceará in 1965 (BOOK1963). (3)Without registration. (4)EMAPA = Empresa Maranhense de Pesquisa Agropecuária. Source: Freire Filho, F.R. Feijão-caupi no Brasil: Produção, melhoramento genético, avanços e desafios. Teresina-PI: Embrapa Meio-Norte, 2011, 84p.
